# Repetitive and restricted behaviours and anxiety in autism spectrum disorder: protocol for a systematic review and meta-analysis

**DOI:** 10.1186/s13643-021-01830-2

**Published:** 2021-12-02

**Authors:** Tegan Sellick, Alexandra Ure, Katrina Williams

**Affiliations:** 1 School of Psychology, Monash University, Wellington Road, Clayton, Victoria 3800 Australia; 2 Department of Paediatrics & Education Research, Monash University, 246 Clayton Road, Clayton, Victoria Australia; 3 Monash Children’s Hospital, Level 5, 246 Clayton Road, Clayton, Victoria Australia; 4 Neurodevelopment & Disability, Murdoch Children’s Research Institute, Royal Children’s Hospital, 50 Flemington Road, Parkville, Victoria 3168 Australia; 5 Mental Health, Royal Children’s Hospital, 50 Flemington Road, Parkville, Victoria 3168 Australia

**Keywords:** Quantitative review, Metanalysis, Asperger’s, Worry, Obsessive-compulsive disorder, Hyperreactivity to sensory input, Hyporeactivity to sensory input

## Abstract

**Background:**

Autism spectrum disorder (ASD) is a neurodevelopmental disorder defined by persistent deficits in social functioning and the presence of restricted and repetitive behaviours (RRBs). RRBs refer to four subtypes of behaviour including repetitive movements, speech, or use of objects; insistence on sameness; restricted interests; and sensory processing abnormalities. Many individuals with ASD also experience anxiety, which compounds ASD-related difficulties and inhibits daily functioning. RRBs have been found to be positively associated with anxiety; however, our understanding of the interplay between RRB subtypes and anxiety remains unclear. Thus, the current review aims to clarify the association between RRBs and anxiety by conducting a systematic review and meta-analysis.

**Methods:**

To identify relevant studies, we will search five databases: CINAHL Plus, Cochrane Central Register of Controlled Trials, Ovid MEDLINE, PsycINFO, and Scopus. Articles included in the review will have their titles, abstracts, and full texts reviewed by two independent authors and their methodological quality assessed via the modified Newcastle-Ottawa Scale. Random-effects meta-analyses will then be conducted to calculate the pooled association between RRB subtypes and anxiety. Sensitivity analyses will also be conducted to assess the potential impact of bias, missing data, outliers, and methodological differences on this relationship. Additionally, this review will collate the factors which may influence the anxiety-RRB relationship to help identify who is most vulnerable to developing anxiety.

**Discussion:**

This will be the first review to examine the association between the four subtypes of RRBs and anxiety in individuals with ASD. Understanding this relationship, and the factors associated with this, may help clinicians understand the different underpinnings and presentations of anxiety within this population with potential implications for assessment and treatment.

**Systematic review registration:**

PROSPERO CRD42020185434

**Supplementary Information:**

The online version contains supplementary material available at 10.1186/s13643-021-01830-2.

## Background

Autism spectrum disorder (ASD) is a neurodevelopmental disorder defined by persistent deficits in social functioning and the presence of restricted and repetitive behaviours (RRBs) [1]. RRBs refer to four subtypes of behaviour including repetitive movements, speech, or use of objects; insistence on sameness; restricted interests; and sensory processing abnormalities [1]. Additionally, many individuals with ASD experience elevated levels of anxiety compared to the general population with approximately 40% of children with ASD meeting the criteria for an anxiety disorder [2] and others displaying subclinical symptoms [3]. The presence of anxiety negatively impacts one’s quality of life by inhibiting daily functioning and increasing the risk of comorbid medical and psychiatric conditions [4]. These deficits are further compounded in individuals with ASD as anxiety can be triggered by or exacerbate the social difficulties inherent in the disorder resulting in significant distress and relationship difficulties with family members, teachers, and peers [5, 6].

Despite these adverse effects, diagnosing and treating anxiety in individuals with ASD is challenging. This is due, in part, to the overlap that exists between anxiety symptoms and autistic traits with the core features of ASD—social communication deficits and RRBs—closely resembling the symptoms of social anxiety and obsessive-compulsive disorder (OCD) respectively [7]. The diagnosis and treatment of anxiety are further complicated by its atypical presentation in individuals with ASD. Anticipatory worry related to changes in routine, sensory issues, unusual fears, and social aversion that is not linked to fears of rejection are often experienced by individuals with ASD alongside traditional anxiety symptoms [8]. This atypical presentation, paired with overlapping symptomology, may result in anxiety being incorrectly diagnosed or remaining undetected and subsequently untreated [9]. Either is potentially detrimental, as anxiety symptoms in youth significantly predict later anxiety disorders and subsequently are a priority for intervention [10]. A diagnosis of anxiety would also direct the type of intervention. As the efficacy of interventions varies between mental disorders and participant characteristics, a diagnosis of anxiety would enable clinicians to select an appropriate intervention [11].

### RRBs and anxiety

There is a body of evidence highlighting the association between RRBs and anxiety symptoms in individuals with ASD [12]. Specifically, cross-sectional studies have found moderate to large correlations between the two constructs (*r* = .41–.69) with individuals experiencing higher anxiety engaging in more RRBs [13–15]. Several longitudinal studies exploring the nature of this relationship have found that the severity of RRBs predicts later anxiety, suggesting that a unidirectional relationship exists between the two constructs (*p* < .001–*p* = 0.026) [12, 16, 17]. The strong and consistent associations found across studies suggest the RRBs and anxiety are interrelated and that the presence of RRB’s precedes the onset of anxiety [12, 16, 17]. Subsequently, RRBs have been conceptualised as an early manifestation of anxiety [12, 16] or a maladaptive response to negative affect which subsequently increases one’s risk of anxiety over time [10, 12, 18]. Although engaging in RRBs may temporarily reduce anxiety symptoms, their continued use may impede one’s ability to adopt more adaptative coping mechanisms, reduce their ability to engage in their environment, and therefore ultimately perpetuate feelings of anxiety [10, 12, 18]. This parallels the trajectory observed in OCD whereby individuals engage in compulsions to neutralise anxiety associated with obsessions [4, 18]. However, in reality, these compulsions add considerable distress to individuals with OCD as they do not address the source of anxiety and interfere with daily functioning [4, 18]. A similar association is seen in posttraumatic stress disorder, where efforts to suppress or avoid memories of an index trauma are helpful in the short term, but lead to long-term increases in intrusive re-experiencing and hyperarousal [19]. As the hypothesised relationship between anxiety and RRBs in ASD is thought to mirror the relationship between obsessions and compulsions in the OCD literature, it is plausible to suggest that RRBs could identify individuals who are vulnerable to experiencing future anxiety. This could help clinicians identify and treat anxiety in young children, preventing the manifestation of significant distress in later years.

### RRB subtypes and anxiety

Although the literature consistently reports that RRBs are associated with anxiety, it is important to clarify if this relationship exists for all or specific RRB subtypes. There is a consistent body of evidence to suggest that insistence on sameness and restricted interests are linked to anxiety [3]. However, the relationship between the remaining subtypes and anxiety remains unclear, with conflicting evidence emerging from the literature. This lack of clarity may be associated with the varying definitions of RRBs. Whilst some studies have explored subtypes individually [3, 20], others have combined the subtypes into two broad domains known as low-order and high-order RRBs [10, 21]. Low-order RRBs refer to repetitive movements that may be motivated by sensory feedback, whereas high-order RRBs include restricted interests and insistence on sameness behaviours [22]. These domains have been criticised for being too broad and therefore disregarding important differences in the functions served by RRB subtypes [3]. The grouping of low-order RRBs may limit our understanding of the relationship between these RRB subtypes and anxiety as they are thought to have contradictory functions. For instance, sensory processing abnormalities may increase one’s risk of experiencing anxiety as having a low threshold for sensory input is thought to exacerbate environmental stressors [17]. This is consistent with the finding that hyperactivity to sensory input in toddlers predicts later anxiety [17]. 

Contrastingly, repetitive movements are thought to enable one to manage anxiety that stems from sensory processing abnormalities [23]. A discrepancy is therefore highlighted between the two low-order RRBs, with one thought to contribute to anxiety, whilst the other a response. 

Subtypes of high-order RRBs are also understood to uniquely moderate anxiety symptoms. For instance, restricted interests are thought to be a coping response to anxiety [18], whilst insistence on sameness behaviours may enable one to control their environment and thus buffer the effect of anxiety [23]. These distinctions are consistent with the results of factor analyses which have found restricted interests and insistence on sameness behaviours to be distinct subtypes [24–26]. Thus, it is critical to examine these subtypes individually in order to clarify their differential relationship with anxiety. This knowledge could help clinicians tailor diagnostic tools and treatment interventions as specific RRBs may be utilised as a marker for anxiety, whilst others could be targeted for interventions.

### The relationship between RRB subtypes

To effectively conceptualise the associations between RRBs and anxiety, it is important to also understand the inter-relationships amongst RRB subtypes. There is emerging evidence to suggest that RRB subtypes are interrelated [23, 27]. Specifically, sensory processing abnormalities have been shown to negatively correlate with repetitive movements and high-order RRBs, whilst a positive association has been observed between repetitive movements and high-order RRBs [23, 27]. However, not all studies yield consistent results with Lidstone et al. [13] finding that low registration (a measure of hyporeactivity) and sensory sensitivity (a measure of hyperreactivity) were not associated with repetitive movements.

### Factors influencing the RRB-anxiety relationship

The relationship between specific RRBs and anxiety may be influenced by the presence of additional RRBs. Black et al. [28] found that hypersensitivity mediated the relationship between anxiety subtypes and high-order RRBs in individuals with ASD. There is additional evidence to suggest that anxiety mediates the relationship between sensory processing abnormalities and high-order RRBs [27]. Drawing from these findings, it is plausible to suggest that restricted interests and insistence on sameness may enable individuals to reduce anxiety associated with sensory input [13, 23]. Additionally, anxiety and intolerance of uncertainty, a trait associated with high worry and avoidance, were found to partially mediate the relationship between sensory processing abnormalities and repetitive movements [23, 27]. These findings suggest that repetitive movements might function to regulate arousal; however, this process may also reduce anxiety [23]. Collating this information may help clinicians understand if these behaviours are interrelated and whether clusters or specific RRBs are associated with anxiety.

Other individual traits or characteristics may also influence the relationship between RRBs and anxiety. For instance, intolerance of uncertainty has been linked to both anxiety and RRBs [22, 23, 29]. Hwang et al. [29] found that intolerance of uncertainty significantly mediated the relationship between anxiety and hyposensitivity, hypersensitivity, and high-order RRBs. Age and intelligence quotient (IQ) have also been identified as factors relating to both anxiety and RRB subtypes [3, 30]; however, the role they play within the RRB-anxiety relationship remains unclear. Findings to date are mixed with some studies finding that anxiety increases with age but not with RRBs [17, 31], whilst others have found the reverse [29]. As all individuals with ASD present with at least two RRBs as per the Diagnostic and Statistical Manual of Mental Disorders (DSM: 1), identifying and collating the factors associated with the RRB-anxiety relationships could help clinicians identify who are most vulnerable to developing anxiety symptoms.

### Current study

Our systematic review aims to (1) meta-analytically assess the association between RRB subtypes and anxiety symptoms in individuals with ASD, (2) meta-analytically assess the association between the RRB subtypes in the context of the anxiety-RRB relationship, and (3) collate factors associated with both RRBs and anxiety symptoms. These aims will be addressed via the following research questions:

1. What is the association between RRB subtypes and anxiety symptoms in people with ASD?
2. In studies exploring RRBs and anxiety in people with ASD, what is the association between the RRB subtypes?
3. For individuals with ASD, what are the factors associated with both RRBs and anxiety?

This review is registered on the PROSPERO database (CRD42020185434).

## Methods

### Eligibility criteria

Observational studies will be included in the review if they meet the following eligibility criteria:

1. The sample consists of participants with a diagnosis of ASD under the DSM (DSM-III, DSM-III-R, DSM-IV, DSM-IV-TR, or DSM-5), International Classification of Diseases (ICD: ICD-9, ICD-10, or ICD-11), or an equivalent diagnosis from the Autism Diagnostic Interview – Revised, Autism Diagnostic Observation Schedule, or Childhood Autism Rating Scale.
2. Studies include at least one standardised measure of anxiety completed by the participant or their parent. This includes measures of anxiety symptoms and disorders as defined by the DSM-5, as well as OCD.
3. Studies include at least one standardised measure of RRBs completed by the participant or their parent.
4. The association between anxiety and RRBs, or an RRB subtype, was analysed or relevant data is reported and available for analysis. If a paper reports that they have analysed the association but do not report the relevant statistics, we will contact the authors and ask them to provide this information.
5. Was published in or after 1987 when RRBs first entered the DSM-III-R criteria for ‘Autistic Disorder’.

Articles will be excluded if they include less than five participants or if they are an opinion piece, editorial, or review. The reference list of relevant reviews will be checked to identify additional research.

### Search strategy

The literature search will be conducted across five databases: CINAHL Plus, Cochrane Central Register of Controlled Trials, Ovid MEDLINE, PsycINFO, and Scopus. The search strategy was developed alongside a senior tertiary librarian to identify studies which meet the above eligibility criteria. The reference list of all eligible articles will also be searched to ensure literature saturation. The search strategy for Ovid MEDLINE is included as an additional file (Additional file 1).

Unpublished materials will also be identified by searching clinical trial registries, ClinicalTrials.gov and Australian New Zealand Clinical Trials Registry (ANZCTR), and thesis databases, Trove and ProQuest Dissertations and Theses Global. The first 50 results of an advanced Google search will also be reviewed to identify papers that were not included in the databases.

The search will be limited to humans.

### Study selection

Articles identified from the literature search will be exported into Endnote X9 and duplicates will be removed.

The titles and abstracts of remaining articles will be imported into Covidence, and their eligibility will be assessed by two independent reviewers. Eligible articles will have this process repeated for the full article. If an article is missing information, the author will be asked to provide this information.

Reviewers will solve disagreements by discussing the matter in relation to the inclusion criteria. If consensus is not reached, a third reviewer will be engaged to resolve this.

A PRISMA flow diagram will highlight the studies remaining at each stage.

### Data extraction

A standardised form will be utilised by two reviewers to extract the following data for each study: (1) study details including the study design; (2) sample size; (3) participant demographics including gender, age, IQ, level of functioning, and severity of ASD symptoms; (4) how ASD diagnoses were confirmed; (5) anxiety type/s investigated; (6) anxiety measure/s used; (7) RRB subtype/s investigated; (8) RRB measure/s used; (9) factors associated with RRBs and anxiety; (10) reported outcomes, statistical analyses, adjustments, and significance; and (11) information for assessing methodological quality (see Additional file 2).

If overlapping studies are identified, we will include the study with the largest sample size to avoid including duplicate samples or studies as this may bias the results.

When data is missing, we will contact the first author of the study via email and ask them to provide this information. If this information is not provided, we will aim to retain the study in the review. However, a sensitivity analysis will be run to assess the impact of missing data on the results.

### Assessing methodological quality

One reviewer will utilise a modified version of the Newcastle-Ottawa Scale to assess the methodological quality of each included study [32]. This scale has been adapted for use with cross-sectional studies to assess selection, comparability, and outcomes (see Additional file 3).

### Data analysis and synthesis

#### Research questions 1 and 2

For each study, the association between anxiety symptoms and RRB subtypes and the association between different RRB subtypes will be reported, including whether the association is unadjusted or adjusted. If the association is adjusted, we will also report the covariates included in the analyses. Categorical (odds ratio (OR) or relative risks (RR)) and continuous (Pearson’s correlation coefficient (*r*), Cohen’s *d*, or outcomes of linear regression) outcomes are expected. If there are two or more studies assessing the association between RRB subtypes and anxiety, or the interplay between RRBs, which utilise different effect sizes, data will be standardised to allow for a more direct comparison [33]. Data will be converted into the effect size which is prominent across the eligible articles.

Homogeneity will be assessed by inspecting the forest plots and *I*^2^ statistic. If the studies are homogenous, a random-effects meta-analysis will be conducted in RStudio using the metacor function to assess the magnitude of the association between anxiety and each RRB subtype or the relationship between the RRB subtypes. Additionally, subgroup analyses will be conducted to investigate important differences based on (1) age, (2) level of functioning, (3) diagnostic characteristics of ASD, (4) IQ or developmental quotient, (5) mental health diagnoses other than anxiety, and (6) anxiety diagnoses.

A sensitivity analysis will also be run to assess the potential impact of (1) methodological differences across studies, (2) methodological quality, (3) missing data across studies, and (4) outliers. To conduct these analyses, the following information will be recorded for each study: (1) the questionnaires utilised by the study, (2) the study's methodological quality (satisfactory/unsatisfactory), (3) if the study is missing data (yes/no), and (4) if the study is an outlier (yes/no). The results of the meta-analysis which includes all studies will then be compared to the results of the meta-analysis which only includes studies with a specific code. For instance, the results of the initial meta-analysis may be contrasted to a meta-analysis which only included studies without missing data to determine the impact of missing data on the results.

Publication bias will be assessed via an examination of funnel plots and Egger’s test.

If there are fewer than four studies for each association of interest, or if studies are not homogenous, we will present a narrative synthesis.

If a study reports unadjusted and adjusted associations, we will pool the unadjusted results as there are no well-validated methods for pooling adjusted results outside of intervention studies.

A meta-regression will be conducted if the data available are suitable.

#### Research question 3: for individuals with ASD, what are the factors associated with both RRBs and anxiety?

We will collate and describe the factors reported to be associated with RRBs and anxiety. Each factor, whether it is significantly associated with the construct, and the associations will be recorded. Whether a factor significantly mediates or moderates the anxiety-RRB relationship will also be reported.

## Discussion

The results of this review will help clarify the association between the RRB subtypes and anxiety. Assessing anxiety in children with ASD is a clinical challenge. By improving our understanding of this relationship and the related interpersonal characteristics, clinicians will be better equipped to understand the underpinnings and presentations of anxiety within this population. We hope this information will lead to improved diagnostic accuracy and tailored interventions based upon individual needs. Specifically, it is possible that specific subtypes of RRBs may be identified and used as an observable marker of anxiety or a point for intervention. Given the current challenges in assessing anxiety in this population, this could help alleviate its long-term psychological and social effects.

## Supplementary Information

**Additional file 1.** OVID Medline Search Strategy.

**Additional file 2.** Data Extraction Form.

**Additional file 3.** Newcastle-Ottawa Quality Assessment.

**Additional file 4.** Amendments to the protocol.

## Abbreviations

ANZCTR: Australian New Zealand Clinical Trials Registry
APA: American Psychiatric Association
ASD: Autism spectrum disorder
DSM: Diagnostic and Statistical Manual of Mental Disorders
ICD: International Classification of Diseases
IQ: Intelligence quotient
OCD: Obsessive-compulsive disorder
OR: Odds ratio
PRISMA-P: Preferred Reporting Items for Systematic Review and Meta-Analysis Protocols
RR: Relative risks
RRBs: Restricted and repetitive behaviours
