# Supplementary material for: Repetitive and restricted behaviours and anxiety in autism spectrum disorder: protocol for a systematic review and meta-analysis
Source: Syst Rev. 2021 Dec 2;10:303. doi: 10.1186/s13643-021-01830-2 (PMC8638349; doi:10.1186/s13643-021-01830-2)
Supplement: Supplementary file 2 — Additional file 2. Data Extraction Form. [file 13643_2021_1830_MOESM2_ESM.docx]

**Additional File 2**

*Data Extraction Form*

| Study Details | |
| --- | --- |
| First author: | |
| Year: |  |
| Location: |  |
| Study Design: |  |
| Sample size: |  |
| Recruited from: |  |
| Primary aim: |  |
| Participant Demographics | |
| Mean age of sample: |  |
| Gender:  (Percentage of males to females) |  |
| Mean IQ of sample: |  |
| Level of functioning:  (measured via the mean of tools such as the Vineland Adaptive Behavior Scales) | |
| Severity of ASD symptoms:  (measured via the mean of tools such as the Autism Diagnostic Observation Schedule) | |
| Comorbid diagnoses: |  |
| Duration of anxiety diagnosis: |  |
| Medications for anxiety or other psychoactive medications: | |
| Previous interventions designed to target anxiety or RRBs: | |
| Method of ASD diagnosis: |  |
| Anxiety Type/s Investigated | |
| Anxiety Measure/s Used | |
| RRB Type/s Investigated | |
| RRB Measure/s Used | |
| Research Question 1 | |
| The association between anxiety and RRB subtypes will be recorded. This will include the outcomes, statistical analyses, adjustments and significance. | |
| Research Question 2 | |
| The association between RRB subtypes will be recorded. This will include the outcomes, statistical analyses, adjustments and significance. | |
| Research Question 3 | |
| Factors identified as being associated with RRBs and anxiety will be recorded. This will include the name of the factor, the statistical analysis utilised to uncover the relationship between the factor and RRBs and anxiety, and the outcome of this analysis (including adjustments and significance). | |
| Information for Assessing Risk of Bias/Methodical Quality | |
