# Supplementary material for: Repetitive and restricted behaviours and anxiety in autism spectrum disorder: protocol for a systematic review and meta-analysis
Source: Syst Rev. 2021 Dec 2;10:303. doi: 10.1186/s13643-021-01830-2 (PMC8638349; doi:10.1186/s13643-021-01830-2)
Supplement: Supplementary file 4 — Additional file 4. Amendments to the protocol. [file 13643_2021_1830_MOESM4_ESM.docx]

**Amendments to the protocol**

The protocol published with PROSPERO had undergone two amendments prior to the submission of this manuscript for publication. The amendments include:

- Searching a second clinical trial database to improve the comprehensiveness of the search;
- Expanding the inclusion criteria to include Obsessive Compulsive Disorder as an anxiety disorder due to its historical link with anxiety;
- Clarifying the inclusion criteria to highlight that all anxiety disorders within the DSM-5 are eligible for this study;
- Removing letters to the editor and articles written in a language other than English from the exclusion criteria as these sources contain valuable data;
- Stating that we will not re-run our search prior to the final analysis as the analysis will take place within one year of the initial search;
- No longer contacting prominent authors in the field searching for articles as we have already executed a comprehensive search strategy;
- Having one reviewer conduct the assessment of methodological quality;
- Having two reviewers conduct data extraction;
- Renaming the field ‘study design’ to ‘study details’ in our data extraction form to capture more information. Additionally, the fields ‘RRB definition’ and ‘RRB subtype/s investigated’ have been merged;
- Including standardised beta from linear regressions as an outcome measure;
- Conducting a sensitivity analysis on any missing data;
- Limiting the advanced Google Search to the first 50 results;
- Clarifying that the Newcastle Ottawa Scale measures methodological quality rather than risk of bias.

Any future amendments to the protocol will also be registered with PROSPERO and will be available online.
